# Thoracic Aortic ^18^F-Sodium Fluoride Activity and Ischemic Stroke in Patients With Established Cardiovascular Disease

**DOI:** 10.1016/j.jcmg.2021.12.013

**Published:** 2022-07

**Authors:** Alexander J. Fletcher, Yong Y. Tew, Evangelos Tzolos, Shruti S. Joshi, Jakub Kaczynski, Jennifer Nash, Samuel Debono, Maria Lembo, Jacek Kwiecinski, Rong Bing, Maaz B.J. Syed, Mhairi K. Doris, Edwin J.R. van Beek, Alistair J. Moss, William S. Jenkins, Niki L. Walker, Nikhil V. Joshi, Tania A. Pawade, Philip D. Adamson, William N. Whiteley, Joanna M. Wardlaw, Piotr J. Slomka, Michelle C. Williams, David E. Newby, Marc R. Dweck

**Affiliations:** a British Heart Foundation Centre for Cardiovascular Science, University of Edinburgh, Edinburgh, United Kingdom; b Department of Advanced Biomedical Sciences, Federico II University of Naples, Naples, Italy; c Department of Interventional Cardiology and Angiology, Institute of Cardiology, Warsaw, Poland; d Edinburgh Imaging Facility, Queens Medical Research Institute, University of Edinburgh, Edinburgh, United Kingdom; e Scottish Adult Congenital Cardiology Service, Golden Jubilee National Hospital, Clydebank, Glasgow, United Kingdom; f Bristol Heart Institute, Bristol Royal Infirmary, University of Bristol, United Kingdom; g Christchurch Heart Institute, University of Otago, Christchurch, New Zealand; h Cedars-Sinai Medical Centre, Department of Imaging (Division of Nuclear Cardiology), Los Angeles, USA; i Centre for Clinical Brain Sciences, UK Dementia Research Institute, University of Edinburgh, Edinburgh, United Kingdom

**Keywords:** calcification, computed tomography, positron emission tomography, ^18^F-sodium fluoride, stroke, thoracic aorta, AUC, area under the curve, CT, computed tomography, PET, positron emission tomography, ROC, receiver-operator characteristic

## Abstract

**Background:**

Aortic atherosclerosis represents an important contributor to ischemic stroke risk. Identifying patients with high-risk aortic atheroma could improve preventative treatment strategies for future ischemic stroke.

**Objectives:**

The purpose of this study was to investigate whether thoracic ^18^F-sodium fluoride positron emission tomography (PET) could improve the identification of patients at the highest risk of ischemic stroke.

**Methods:**

In a post hoc observational cohort study, we quantified thoracic aortic and coronary ^18^F-sodium fluoride activity in 461 patients with stable cardiovascular disease undergoing PET combined with computed tomography (CT). Progression of atherosclerosis was assessed by change in aortic and coronary CT calcium volume. Clinical outcomes were determined by the occurrence of ischemic stroke and myocardial infarction. We compared the prognostic utility of ^18^F-sodium fluoride activity for predicting stroke to clinical risk scores and CT calcium quantification using survival analysis and multivariable Cox regression.

**Results:**

After 12.7 ± 2.7 months, progression of thoracic aortic calcium volume correlated with baseline thoracic aortic ^18^F-sodium fluoride activity (n = 140; r = 0.31; *P =* 0.00016). In 461 patients, 23 (5%) patients experienced an ischemic stroke and 32 (7%) a myocardial infarction after 6.1 ± 2.3 years of follow-up. High thoracic aortic ^18^F-sodium fluoride activity was strongly associated with ischemic stroke (HR: 10.3 [95% CI: 3.1-34.8]; *P =* 0.00017), but not myocardial infarction *(P =* 0.40). Conversely, high coronary ^18^F-sodium fluoride activity was associated with myocardial infarction (HR: 4.8 [95% CI: 1.9-12.2]; *P =* 0.00095) but not ischemic stroke *(P =* 0.39). In a multivariable Cox regression model including imaging and clinical risk factors, thoracic aortic ^18^F-sodium fluoride activity was the only variable associated with ischemic stroke (HR: 8.19 [95% CI: 2.33-28.7], *P* = 0.0010).

**Conclusions:**

In patients with established cardiovascular disease, thoracic aortic ^18^F-sodium fluoride activity is associated with the progression of atherosclerosis and future ischemic stroke. Arterial ^18^F-sodium fluoride activity identifies localized areas of atherosclerotic disease activity that are directly linked to disease progression and downstream regional clinical atherothrombotic events. (DIAMOND–Dual Antiplatelet Therapy to Reduce Myocardial Injury [DIAMOND], NCT02110303; Study Investigating the Effect of Drugs Used to Treat Osteoporosis on the Progression of Calcific Aortic Stenosis [SALTIRE II], NCT02132026; Novel Imaging Approaches To Identify Unstable Coronary Plaques, NCT01749254; and Role of Active Valvular Calcification and Inflammation in Patients With Aortic Stenosis, NCT01358513)

Ischemic stroke remains a leading cause of serious long-term disability and mortality across the world.^1^ Current preventative strategies focus on addressing the underlying causes and modifiable risk factors for stroke.^2^ Comprehensive analysis of multiple large community data sets has allowed the optimization of clinical risk scores providing generalized estimates of stroke risk.^3^^,^^4^ Although these well-validated estimates provide a guide to risk at the epidemiological level, noninvasive imaging has the potential to detect and to quantify disease in a more precise and patient-specific manner. Such information can provide a personalized approach to risk stratification and preventative treatment.

Imaging of the thoracic aorta can directly visualize atheromatous lesions, which have been consistently associated with the risk of ischemic stroke.^5^^,^^6^ Calcified atheromatous lesions of the ascending and arch of the aorta are readily detected and quantified on conventional computed tomography (CT), with calcium scores providing some incremental value for the prediction of future stroke risk.^7^^,^^8^ However, these overtly calcified vascular lesions are thought to represent a later and more stable stage in the disease process. Conversely, arterial ^18^F-sodium fluoride positron emission tomography (PET) identifies an earlier and more active stage of atheromatous disease that is associated with plaque vulnerability and the culprit lesions underlying atherothrombotic events.^9^, ^10^, ^11^ In other cardiovascular conditions, arterial ^18^F-sodium fluoride activity provides an assessment of disease activity that is associated with disease progression and clinical events.^12^ The potential of aortic ^18^F-sodium fluoride activity to assess thoracic aortic atherosclerotic disease progression and to predict downstream clinical outcomes is unknown.^13^^,^^14^

We here assess whether thoracic aortic ^18^F-sodium fluoride activity predicts calcified atheromatous plaque disease progression and whether it can provide important information on the future risk of ischemic stroke or myocardial infarction in patients with established cardiovascular disease.

## Methods

### Study populations

In this post hoc analysis, the study population comprised patients with a primary diagnosis of stable coronary artery disease or aortic stenosis who had undergone ^18^F-sodium fluoride PET-CT of the thoracic aorta in 1 of 4 prospective clinical imaging studies conducted at a single institution (see the Supplemental Methods for full details of inclusion criteria, randomization, and study outcomes). Two randomized controlled trials: the DIAMOND (Dual Antiplatelet Therapy to Reduce Myocardial Injury) study (stable multivessel coronary artery disease, n = 220, NCT02110303); and the SALTIRE II (Study Investigating the Effect of Drugs Used to Treat Osteoporosis on the Progression of Calcific Aortic Stenosis) (mild to severe aortic stenosis, n = 199, NCT02132026), as well as 2 observational cohort studies: the Novel Imaging Approaches to Identify Unstable Coronary Plaques study (stable angina undergoing angiography or acute myocardial infarction, n = 80, NCT01749254); and Role of Active Valvular Calcification and Inflammation in Patients With Aortic Stenosis (n = 121, NCT01358513). Those with acute myocardial infarction or aortic sclerosis, or control subjects without coronary or aortic valve disease were excluded (see Supplemental Figure 1 for CONSORT diagram). The principal findings of these studies have been reported previously, with both randomized controlled trials reporting no difference in the primary outcome between treatment and placebo groups.^9^^,^^15^, ^16^, ^17^ Demographics, clinical risk factors, and history of cardiovascular disease were recorded, and 10-year revised Framingham stroke risk score was calculated for each patient.^3^ This study complies with the Declaration of Helsinki, with each of the studies approved by regional ethical committees and written, informed consent provided by each participant.

### PET-CT image acquisition protocol

All scans were performed in a single image acquisition session, 60 minutes after injection of 125-250 MBq of ^18^F-sodium fluoride on a hybrid PET-CT scanner (128-multidetector Biograph mCT, Siemens Medical Systems) at a single center. Attenuation correction CT was performed immediately before PET data acquisition (100-120 kV, current 40-50 mA) and was reconstructed at 3-mm slice thickness. The field of view incorporated the heart and whole thoracic aorta including the first branches of the head and neck vessels. PET data were acquired with electrocardiography gating in list-mode during a single 30-minute bed position. PET images were reconstructed into 4 cardiac phases. All PET image reconstructions were performed using an ultra-high-definition algorithm, which applies point-spread function and time-of-flight techniques on a 256 × 256 matrix (109 slices, slice thickness 2.027 mm) using 2 iterations, a 5-mm Gauss filter, and 21 subsets.

### PET-CT image analysis

All PET image analysis was conducted using FusionQuant version 1.20 (Cedars-Sinai Medical Center) blind to clinical characteristics and outcomes. Thoracic aortic ^18^F-sodium fluoride activity was calculated in each patient as described previously.^13^ Briefly, using noncontrast CT and ^18^F-sodium fluoride PET images, a center line extending from the sinotubular junction to the point immediately distal to the left subclavian artery was drawn with a diameter set to the maximal luminal diameter of the aorta +4 mm, accounting for the spatial resolution of PET imaging. The concentration of ^18^F-sodium fluoride activity (SUV/cm^3^) was divided by the mean background activity (SUV/cm^3^) in left and right atria to give the thoracic aortic ^18^F-sodium fluoride activity (Supplemental Figure 2).

Coronary ^18^F-sodium fluoride activity is a reproducible method of quantifying the cumulative ^18^F-sodium fluoride activity in the main epicardial coronary arteries over a 95% threshold of background activity and has been reported previously in the stable coronary artery and stable angina cohorts, but not the aortic stenosis (SALTIRE II) cohort, which is reported here for the first time.^18^^,^^19^ Patients without contrast CT coronary angiography were excluded as it is not possible to quantify coronary ^18^F-sodium fluoride activity from a noncontrast CT. Blood clearance correction and motion correction fused to the third (diastolic) gate were applied to improve the accuracy of coronary ^18^F-sodium fluoride activity.^20^^,^^21^

### CT calcium scores and disease progression

Calcium scores, calcium volume and calcium mass were calculated across the ascending aorta and aortic arch on the attenuation correction CT scans for each patient using OsiriX version 12.0.0 (Bernex) as described previously.^22^ To allow for direct comparison to thoracic aortic ^18^F-sodium fluoride activity, thoracic aortic calcium volume, mass, and scores were calculated from the sinotubular junction to the point immediately distal to the left subclavian artery. Coronary calcium scores were also calculated across the coronary arteries using dedicated gated noncontrast CT calcium score scans. In a subset of patients who had follow-up attenuation CT at ≥6 months, thoracic ascending aortic calcium volume, mass, and scores were measured on the follow-up scans, and the annualized rate of calcium progression was determined.

### Clinical follow-up

Ischemic stroke or myocardial infarction events were ascertained up to the December 31, 2020, from electronic medical records and time-to-event from baseline assessment was calculated. Stroke events were identified as radiologically confirmed cortical infarcts reported by blinded radiologist, or a clinical diagnosis and classification of stroke recorded by the attending physicians independent of the research team and without knowledge of the ^18^F-sodium fluoride activity. Similarly, myocardial infarction events were identified based on clinical diagnosis recorded by the attending cardiologist blinded to the ^18^F-sodium fluoride activity. Clinical characteristics of the stroke events were collated, including symptomology, management, stroke territory subcategory (Bamford classification), and neurovascular imaging results (carotid ultrasound, CT or magnetic resonance brain imaging). Because of the potential for diagnostic misclassification or inclusion of nonatherothromboembolic etiologies, transient ischemic attacks without radiologically confirmed cortical infarcts and lacunar strokes were excluded.^23^ Clinical details of myocardial infarction for the stable coronary artery cohorts have been reported previously.^18^

### Statistical analysis

Categorical variables were presented as number (percentage). Continuous variables with normal distribution were presented as mean ± SD, whereas non-normally distributed variables were presented as median (interquartile interval). Analyses of variable influence on thoracic aortic ^18^F-sodium fluoride activity and disease progression were performed using Pearson’s or Spearman’s correlation, Student’s *t*-test, analysis of variance, Wilcox, or Kruskal-Wallis tests as appropriate. For correlation or regression analysis, variables not normally distributed (aortic calcium score, coronary calcium score and coronary ^18^F-sodium fluoride activity) were log transformed after adding +0.01 (coronary ^18^F-sodium fluoride activity, range 0-25) or +1 (aortic and coronary calcium scores). Receiver-operating characteristic (ROC) analysis for the outcome of stroke was performed for revised Framingham 10-year stroke risk, thoracic aortic calcium score, and thoracic aortic ^18^F-sodium fluoride activity. The optimal threshold for thoracic aortic ^18^F-sodium fluoride activity was determined by Youden’s J statistic, whereas the coronary ^18^F-sodium fluoride activity threshold of ≥1.56 was used as reported previously, because the derivation cohort for this threshold overlaps with the present cohort.^18^ Cumulative and dynamic time-dependent ROC curves and estimated areas under the curve (AUCs) were calculated for yearly time points up to 5 years. The difference between estimated AUCs as well as the variance of the difference using the independent and identically distributed representations of the AUC estimators at each timepoint for revised Framingham 10-year stroke risk and thoracic aortic calcium score were compared with those for thoracic aortic ^18^F-sodium fluoride activity. For event-free survival analysis, separate Kaplan-Meier estimation and cumulative incidence for stroke and myocardial infarction were assessed for both thoracic aortic ^18^F-sodium fluoride activity and coronary ^18^F-sodium fluoride activity using the previously mentioned thresholds. Univariable Cox regression analysis was performed to assess the relationship between coronary and thoracic aortic calcium scores, thoracic and coronary aortic ^18^F-sodium fluoride activity, clinical factors included in the revised Framingham stroke risk model, and stroke outcome. Multivariable Cox regression models included the revised Framingham stroke risk model, thoracic aortic calcium score, and thoracic aortic ^18^F-sodium fluoride activity, both as a continuous or binary variable, which were associated with stroke risk on univariable analysis. Statistical significance was taken as a 2-sided *P ≤* 0.05. All statistical analyses were performed in the open-source statistical software package R (version 4.0.2).

## Results

The final study cohort comprised 461 patients with advanced stable coronary artery disease or aortic stenosis followed up for a mean of 6.1 ± 2.3 years (Supplemental Figure 2). The study population had a high prevalence of cardiovascular risk factors and prior cardiovascular disease (Table 1). Most patients were on preventative therapies (76% antiplatelet therapy, 7% anticoagulant therapy, 79% statin therapy, and 88% antihypertensive therapy). Based on revised Framingham stroke risk, a mean of 15% ± 9% of patients were expected to have a stroke within 10 years. Revised Framingham stroke risk was similar across the individual study cohorts *(P =* 0.54) (Table 1).^3^

**Table 1:** Clinical Characteristics of the Study Population

	Overall (N = 461)	Stable Coronary Artery Disease: Observational Cohort Study (NCT01749254) (n = 38)	Stable Coronary Artery Disease: Randomized Controlled Trial (DIAMOND) (NCT02110303) (n = 201)	Aortic Stenosis: Randomized Controlled Trial (SALTIRE II) (NCT02132026) (n = 158)	Aortic Stenosis: Observational Cohort Study (NCT01358513) (n = 64)
Age, y	69.98 ± 8.48	67.37 ± 8.27	67.66 ± 8.40	72.67 ± 7.77	72.21 ± 8.10
Male	363 (78.7)	34 (89.5)	162 (80.6)	125 (79.1)	42 (65.6)
White ethnicity	394 (99.2)	37 (97.4)	200 (99.5)	156 (98.7)	NR
Body mass index, kg/m^2^	29.60 ± 5.10	29.99 ± 4.54	29.69 ± 5.26	30.05 ± 5.28	27.85 ± 4.07
Current smoker	54 (11.7)	5 (13.2)	29 (14.4)	13 (8.2)	7 (10.9)
Diabetes mellitus	87 (19.0)	3 (7.9)	37 (18.4)	37 (23.4)	10 (15.9)
Hypertension	310 (67.2)	34 (89.5)	114 (56.7)	120 (75.9)	42 (65.6)
Systolic pressure, mm Hg	146.05 ± 19.26	135.24 ± 13.80	146.09 ± 19.78	148.97 ± 19.26	144.3 ± 18.19
Diastolic pressure, mm Hg	78.83 ± 11.41	77.52 ± 10.34	80.44 ± 10.74	77.22 ± 11.55	78.42 ± 13.08
Hypercholesterolemia	363 (78.9)	37 (97.4)	194 (96.5)	97 (61.4)	35 (55.6)
Total cholesterol, mmol/L	4.35 ± 1.08	3.90 ± 0.80	4.21 ± 0.98	4.39 ± 1.01	5.00 ± 1.36
Atrial fibrillation	26 (5.7)	1 (2.6)	5 (2.5)	12 (7.6)	8 (12.5)
Ischemic heart disease	317 (68.8)	38 (100.0)	201 (100.0)	59 (37.3)	19 (29.7)
Previous myocardial infarction	178 (38.8)	13 (34.2)	142 (70.6)	18 (11.4)	5 (8.1)
Previous coronary artery bypass graft	71 (15.4)	11 (28.9)	40 (19.9)	17 (10.8)	3 (4.8)
Previous percutaneous coronary intervention	226 (49.0)	19 (50.0)	163 (81.1)	34 (21.5)	10 (15.6)
Previous transient ischemic attack/stroke	29 (6.3)	4 (10.5)	4 (2.0)	16 (10.1)	5 (8.1)
Creatinine, μmol/L	83.75 ± 20.29	85.65 ± 23.45	80.35 ± 15.26	84.13 ± 19.52	92.36 ± 29.68
Hypertension treatment	405 (87.9)	36 (94.7)	190 (94.5)	131 (82.9)	48 (75.0)
Angiotensin-converting enzyme inhibitor	230 (49.9)	14 (36.8)	132 (65.7)	60 (38.0)	24 (37.5)
Angiotensin receptor blocker	68 (14.8)	4 (10.5)	29 (14.4)	28 (17.7)	7 (11.1)
Thiazide diuretic	75 (16.3)	2 (5.3)	20 (10.0)	33 (20.9)	20 (31.7)
Calcium-channel blocker	114 (24.8)	16 (42.1)	39 (19.4)	47 (29.7)	12 (19.0)
Beta-blocker	241 (52.4)	27 (71.1)	135 (67.2)	57 (36.1)	22 (34.9)
Antiplatelet treatment	348 (75.5)	33 (86.8)	195 (97.0)	84 (53.2)	36 (56.2)
Aspirin	325 (70.7)	31 (81.6)	195 (97.0)	65 (41.1)	35 (54.0)
Clopidogrel	39 (8.5)	4 (10.5)	7 (3.5)	25 (15.8)	3 (4.7)
Ticagrelor	101 (21.9)	0 (0.0)	100 (50.3)	1 (0.6)	0 (0.0)
Anticoagulation treatment	32 (7.0)	3 (7.9)	1 (0.5)	24 (15.2)	4 (6.3)
Vitamin K antagonist	25 (5.4)	3 (7.9)	0 (0.0)	18 (11.4)	4 (6.3)
Direct oral anticoagulant	11 (2.4)	0 (0)	1 (0.5)	10 (6.3)	0 (0.0)
Statin	366 (79.4)	34 (89.5)	183 (91.0)	112 (70.9)	37 (57.8)
Revised 10-y Framingham stroke risk, %	15 ± 9	13 ± 7	15 ± 9	16 ± 9	15 ± 12

Values are n (%) or mean ± SD, unless otherwise indicated.

DIAMOND = Dual Antiplatelet Therapy to Reduce Myocardial Injury; SALTIRE II = Study Investigating the Effect of Drugs Used to Treat Osteoporosis on the Progression of Calcific Aortic Stenosis; NR = not recorded.

### Computed tomography and positron emission tomography

There was a wide range of thoracic aortic calcium scores (230 [17-901]), although 75 (16%) patients had no calcification of the thoracic aorta (Table 2). Thoracic aortic ^18^F-sodium fluoride activity was moderately correlated with log aortic calcium score (Pearson’s r = 0.39; *P* < 0.0001) and was weakly associated with age (Pearson’s r = 0.29; *P* < 0.0001), systolic blood pressure (Pearson’s r = 0.15; *P =* 0.0012) and revised Framingham stroke risk (Pearson’s r = 0.21; *P* < 0.0001). Patients with aortic stenosis had slightly higher aortic calcium scores than those without (595 vs 111 Agatston units; *P* < 0.0001) and higher mean thoracic aortic ^18^F-sodium fluoride activity (1.096 vs 1.069; *P =* 0.0026), although there was no association between these assessments and the degree of aortic stenosis severity (continuous Pearson’s *P =* 0.32; categorical Kruskal Wallis *P =* 0.31). Women had slightly higher thoracic aortic ^18^F-sodium fluoride activity than men (1.09 ± 0.10 vs 1.08 ± 0.09; *P =* 0.021). There were no associations among thoracic aortic ^18^F-sodium fluoride activity and total serum cholesterol concentration, smoking status, atrial fibrillation, hypertension, or diabetes mellitus (all *P >* 0.05).

**Table 2:** Computed Tomography and Positron Emission Tomography Findings

	Overall (N = 461)	Stable Coronary Artery Disease: Observational Cohort Study (NCT01749254) (n = 38)	Stable Coronary Artery Disease: Randomized Controlled Trial (DIAMOND) (NCT02110303) (n = 201)	Aortic Stenosis: Randomized Controlled Trial (SALTIRE II) (NCT02132026) (n = 158)	Aortic Stenosis: Observational Cohort Study (NCT01358513) (n = 64)
Thoracic aortic calcium volume, mL	761 (98-2,423)	517 (6-1,407)	328 (23-1,285)	1,793 (426-3,880)	1,323 (163-2,739)
Ascending calcium volume	0 (0-0)	0 (0-0)	0 (0-0)	0 (0-58)	0 (0-29)
Arch calcium volume	720 (89-2,282)	516 (6-1,406)	324 (23-1,193)	1,683 (389-3,562)	1,323 (157-2,669)
Thoracic aortic calcium mass, g	669 (72-2,547)	423 (4-1,722)	272 (15-1,235)	1,733 (352-3,220)	1,083 (146-2,854)
Ascending calcium mass	0 (0-0)	0 (0-0)	0 (0-0)	0 (0-38)	0 (0-17)
Arch calcium mass	667 (62-2,471)	423 (4-1,722)	259 (15-1,236)	1,710 (310-3,770)	1,083 (112-2,832)
Thoracic aortic calcium score, AU	230 (17-901)	101 (0.75-549)	111 (3-473)	670 (116-1,487)	322 (32-955)
Ascending calcium score, AU	0 (0-0)	0 (0-0)	0 (0-0)	0 (0-7)	0 (0-4)
Arch calcium score, AU	222 (14-865)	101 (0.75-549)	104 (3-470)	661 (98-1,425)	322 (23-951)
Thoracic aortic ^18^F-sodium fluoride activity (unitless)	1.08 ± 0.10	1.05 ± 0.08	1.07 ± 0.08	1.09 ± 0.10	1.10 ± 0.12
Ascending aorta (unitless)	1.07 ± 0.09	1.05 ± 0.08	1.07 ± 0.08	1.07 ± 0.09	1.09 ± 0.11
Arch of the aorta (unitless)	1.12 ± 0.13	1.08 ± 0.10	1.11 ± 0.11	1.14 ± 0.14	1.12 ± 0.15
Coronary calcium score, AU	853 (122-1,105)	579 (91-1,217)	383 (114-902)	575 (140-1,480)	NA
Coronary ^18^F-sodium fluoride activity	0.60 (0.00-2.79)	0.82 (0.00-3.05)	0.34 (0.00-2.64)	0.73 (0.00-2.76)	NA

Values are mean ± SD or median (IQR).

AU = Agatston units; NA = not available; other abbreviations as in Table 1.

### Disease progression

**Figure 1:**
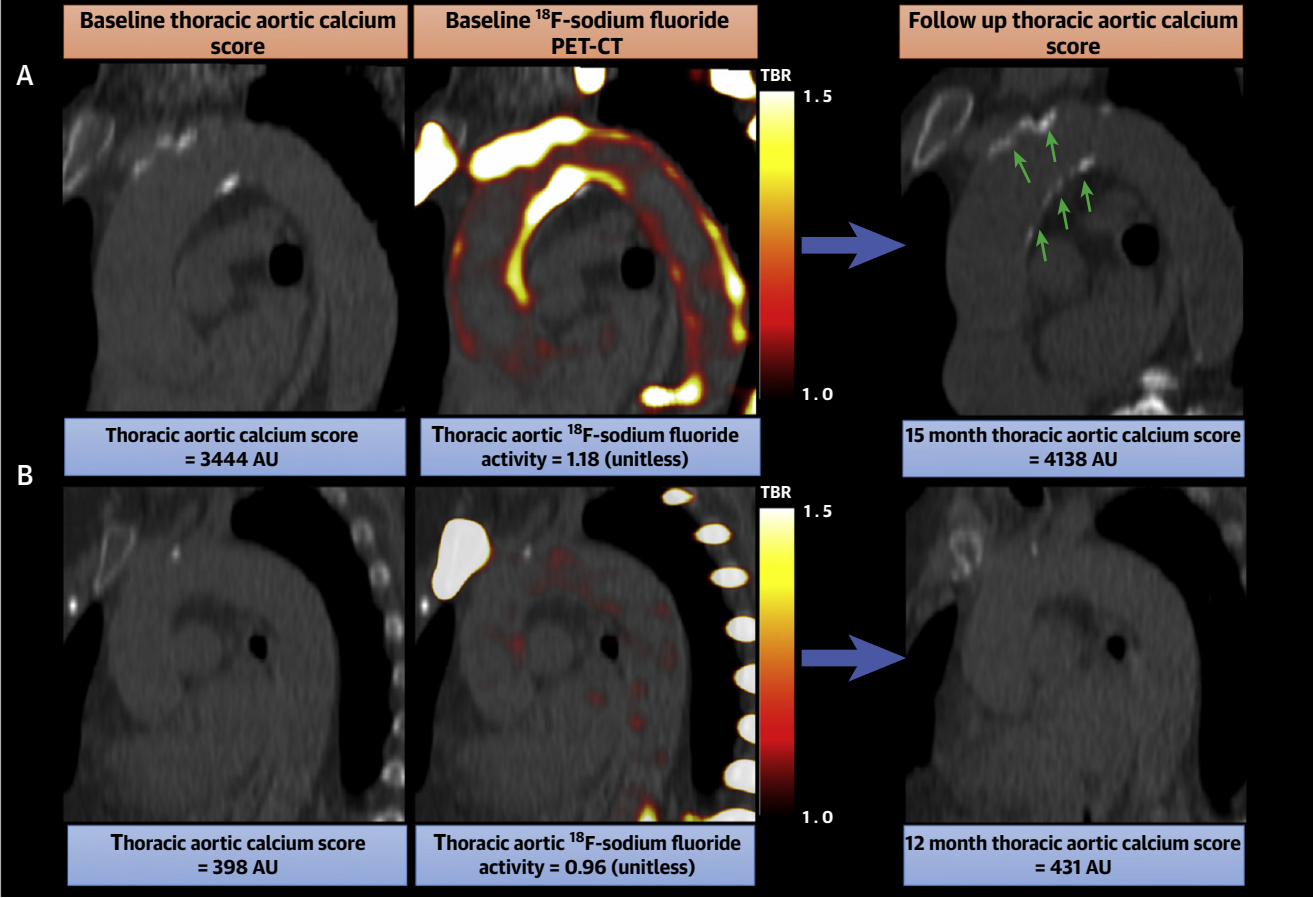
Thoracic Aortic ^18^F-Sodium Fluoride Activity and Progression of Aortic Calcification Relationship between thoracic aortic ^18^F-sodium fluoride activity and progression of thoracic aortic calcium score (Agatston units [AU]). **(A)** Example case in which intense ^18^F-sodium fluoride activity on positron emission tomography and computed tomography (PET-CT) precedes areas of macrocalcification on computed tomography. **(B)** Example case of low ^18^F-sodium fluoride activity and minimal progression of macrocalcification on computed tomography. TBR = tissue-to-background ratio.

Of those undergoing repeat CT, thoracic aortic and coronary calcium score progression could be calculated in 140 (107 with aortic stenosis and 33 with coronary artery disease) and 231 (72 with aortic stenosis and 159 with coronary artery disease) patients, respectively. The median annualized change in thoracic aortic calcium score was 82 AU/y (9-187 AU/y) with a maximum of 1,489 AU/y. The median annualized change in coronary calcium score was 89 AU/y (18-190 AU/y) with a maximum of 1,352 AU/y. Thoracic aortic ^18^F-sodium fluoride activity correlated with the progression of thoracic aortic calcium volume (Pearson’s r = 0.31; *P* = 0.00016), mass (Pearson’s r = 0.29; *P =* 0.00042) (Figure 1), and calcium score (Pearson’s r = 0.23; *P =* 0.0054) as well as coronary calcium score progression (Pearson’s r = 0.25; *P =* 0.012). Coronary ^18^F-sodium fluoride activity did not correlate with progression of aortic calcium score, volume, or mass (Pearson’s all *P >* 0.80) but was moderately correlated with coronary calcium score progression (Pearson’s r = 0.43; *P* < 0.0001).

### Clinical events

Over a mean of 6.1 ± 2.3 years of follow-up, 23 of 461 (5.0%) patients experienced the outcome of ischemic stroke, representing an overall incidence of 9.2 per 1,000 patient-years (Table 3). Of these, 3 (13.6%) had a carotid artery stenosis of >70% and 9 (39.1%) were in atrial fibrillation, of whom 6 were on anticoagulation at the time of stroke (Supplemental Table 1). Over the same period, 32 (6.9%) patients experienced myocardial infarction, representing an overall incidence of 13.5 per 1,000 patient-years.

**Table 3:** Stroke and Myocardial Infarction

	Overall (N = 461)	Stable Coronary Artery Disease: Observational Cohort Study (NCT01749254) (n = 38)	Stable Coronary Artery Disease: Randomized Controlled Trial (DIAMOND) (NCT02110303) (n = 201)	Aortic Stenosis: Randomized Controlled Trial (SALTIRE II) (NCT02132026) (n = 158)	Aortic Stenosis: Observational Cohort Study (NCT01358513) (n = 64)
Mean follow-up, y	6.1 ± 2.3	8.0 ± 2.0	4.6 ± 0.9	4.0 ± 0.9	8.2 ± 2.8
Total patient-y follow-up	2,393	305	929	626	533
Stroke events	23 (5.0)	4 (10.5)	5 (2.5)	7 (4.4)	7 (10.9)
Stroke incidence (per 1,000 patient-y)	9.2	13.1	5.4	11.2	11.3
Stroke subtype (Bamford Classification)					
Total anterior circulation infarct	0	0	0	0	1
Partial anterior circulation infarct	9	3	3	3	4
Posterior circulation infarct	7	1	2	4	2
Significant carotid disease	3/17	1/4	1/5	0/6	1/2
Myocardial infarction events	32 (6.9)	6 (15.7)	13 (7.0)	6 (4.0)	7 (11.0)
Myocardial infarction incidence (per 1,000 patient-y)	13.5	20.0	14.3	9.5	13.2
ST-segment elevation myocardial infarction	3	1	2	0	2
Non-ST-segment elevation myocardial infarction	22	5	11	6	5

Values are n (%) or mean ± SD.

Abbreviations as in Table 1.

Thoracic aortic ^18^F-sodium fluoride activity was higher in those experiencing ischemic stroke than those without stroke (1.15 ± 0.05 vs 1.08 ± 0.10, respectively; *P =* 0.00020) (Table 4). Higher thoracic aortic calcium score (712 [343-1,013] vs 210 [13-871]; *P =* 0.012) (Table 4), but not coronary calcium score *(P =* 0.15) or coronary ^18^F-sodium fluoride activity *(P =* 0.36), was associated with stroke.

**Table 4:** Clinical and Imaging Characteristics Associated With Stroke

	No Stroke (n = 438)	Stroke (n = 23)	*P* Value
Clinical factors			
Revised Framingham 10-y stroke risk, %	15 ± 9	18 ± 8	0.068
Age, y	69.8 ± 8.5	74.0 ± 7.4	0.02
Male	343 (78.7)	18 (78.3)	1.00
Atrial fibrillation	23 (5.3)	3 (13.0)	0.27
Diabetes	83 (19.1)	3 (13.0)	0.64
Systolic blood pressure, mm Hg	146 ± 19	150 ± 21	0.36
Hypertension medication	385 (88.3)	18 (78.3)	0.26
Current smoker	51 (11.7)	2 (8.7)	0.9
Antiplatelet therapy	326 (74.8)	20 (87.0)	0.29
Anticoagulation therapy	28 (6.4)	4 (17.4)	0.11
Imaging biomarkers			
Thoracic aortic ^18^F-sodium fluoride activity, unitless	1.08 ± 0.10	1.15 ± 0.05	0.0002
Ascending aorta, unitless	1.07 ± 0.09	1.14 ± 0.05	0.00012
Aortic arch, unitless	1.11 ± 0.13	1.19 ± 0.07	0.004
Thoracic aortic calcium score, AU	210 (13-871)	712 (343-1,013)	0.012
Ascending calcium score, AU	0 (0-0)	0 (0-14)	0.0073
Arch calcium score, AU	207 (13-838)	691 (298-948)	0.045
Thoracic aortic calcium volume, AU	705 (88-2,325)	1,956 (858-2,607)	0.012
Ascending calcium volume, AU	0 (0-0)	0 (0-116)	0.0092
Arch calcium volume, AU	661 (78-2,219)	1,799 (716-2,569)	0.043
Thoracic aortic calcium mass, AU	614 (59-2,545)	2,027 (902-2,667)	0.012
Ascending calcium mass, AU	0 (0-0)	0 (0-80)	0.0091
Arch calcium mass, AU	582 (56-2,481)	1,938 (792-2,442)	0.04
Coronary ^18^F-sodium fluoride activity^a^	0.60 (0-2.79)	1.4 (0-3.72)	0.36
Coronary calcium score, AU^a^	452 (112-1,075)	1,057 (197-1,888)	0.15

Values are mean ± SD, n (%), or median (IQR).

AU = Agatston units.

a No stroke (n = 381), stroke (n = 1).

**Figure 2:**
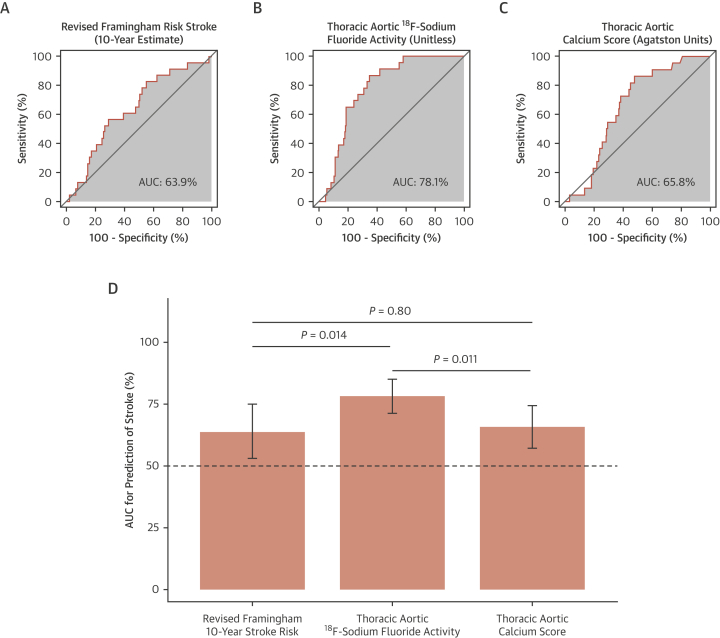
Receiver-Operating Characteristic Curves for Prediction of Stroke Area under the curve (AUC) for **(A)** 10-year stroke clinical risk score, **(B)** thoracic aortic ^18^F-sodium fluoride activity, and **(C)** thoracic aortic calcium score for the outcome of stroke. **(D)** AUC analysis comparing thoracic aortic ^18^F-sodium fluoride activity, clinical risk score, and thoracic aortic calcium score.

**Figure 3:**
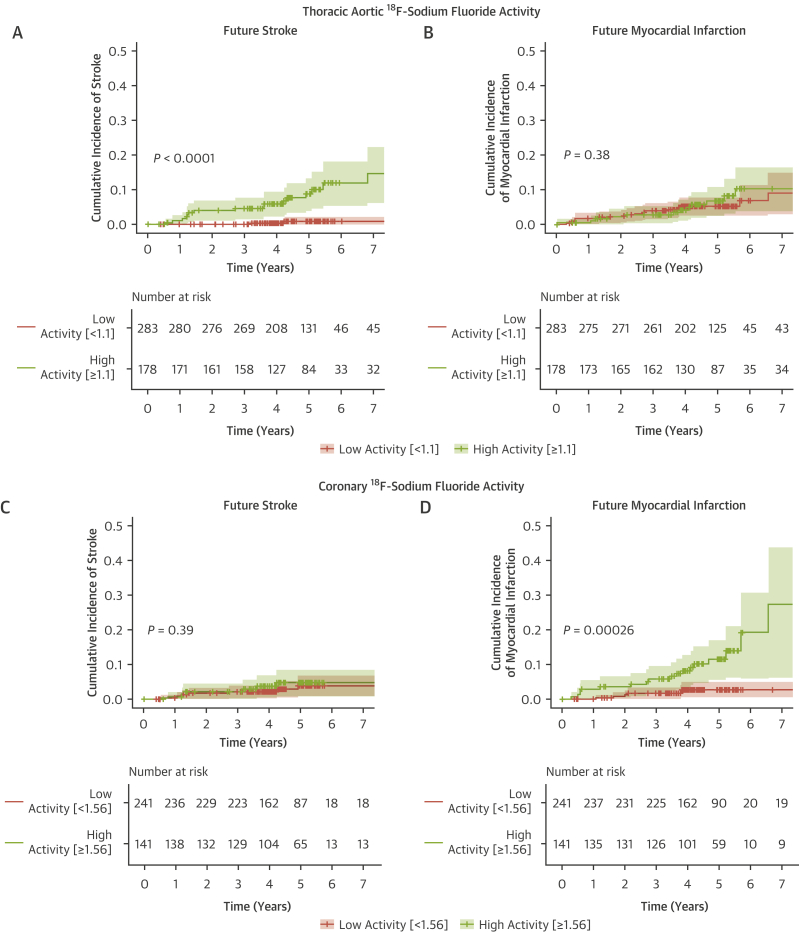
Aortic and Coronary ^18^F-Sodium Fluoride Activity and the Risk of Stroke and Myocardial Infarction Cumulative incidence curves demonstrating freedom from stroke **(A and C)** or myocardial infarction **(B and D)** across the combined cohort. **(A)** Thoracic aortic ^18^F-sodium fluoride activity threshold of ≥1.1 (n = 461) is strongly associated with future stroke (HR: 10.3 [95% CI: 3.1-34.8]; *P =* 0.00017), **(B)** but not myocardial infarction (HR: 1.35 [95% CI: 0.67-2.7]; *P =* 0.40). **(C)** Coronary ^18^F-sodium fluoride activity (n = 382, threshold 1.56)^18^ is not associated with future stroke (HR: 1.59 [95% CI: 0.56-4.53] *P =* 0.39) **(D)** but is strongly associated with future myocardial infarction (HR: 4.8 [95% CI: 1.9-12.2]; *P =* 0.00095). *P* values represent log-rank test.

ROC curves demonstrated thoracic aortic ^18^F-sodium fluoride activity to be a better discriminator of future stroke than thoracic aortic calcium score and the revised Framingham 10-year stroke risk (Figure 2). This finding was consistent in a sensitivity analysis excluding patients with atrial fibrillation or stenotic carotid disease (Supplemental Figure 4). The optimal threshold for thoracic aortic ^18^F-sodium fluoride activity was ≥1.1, which conferred a sensitivity of 87% and specificity of 65% for the prediction of future ischemic stroke. Time-dependent ROC curves demonstrated that AUC for thoracic aortic ^18^F-sodium fluoride activity was a better discriminator of future stroke than thoracic aortic calcium score and the revised Framingham 10-year stroke risk, although this advantage was reduced at 5 years (Supplemental Figure 3). Patients with thoracic aortic ^18^F-sodium fluoride activity ≥1.1 had a 10-fold higher cumulative incidence of stroke than those with thoracic aortic ^18^F-sodium fluoride activity <1.1 (HR: 10.3 [95% CI: 3.06-34.8]; *P =* 0.00017) (Figure 3). The relationship between ^18^F-sodium fluoride activity and event risk appeared to be region-specific, with thoracic aortic ^18^F-sodium fluoride activity being able to identify those at increased risk of ischemic stroke but not myocardial infarction *(P =* 0.40), whereas coronary ^18^F-sodium fluoride activity identified those at increased risk of myocardial infarction (HR: 4.81 [95% CI: 1.89-12.2]; *P =* 0.00095), but not ischemic stroke *(P =* 0.39) (Figure 3).

**Figure 4:**
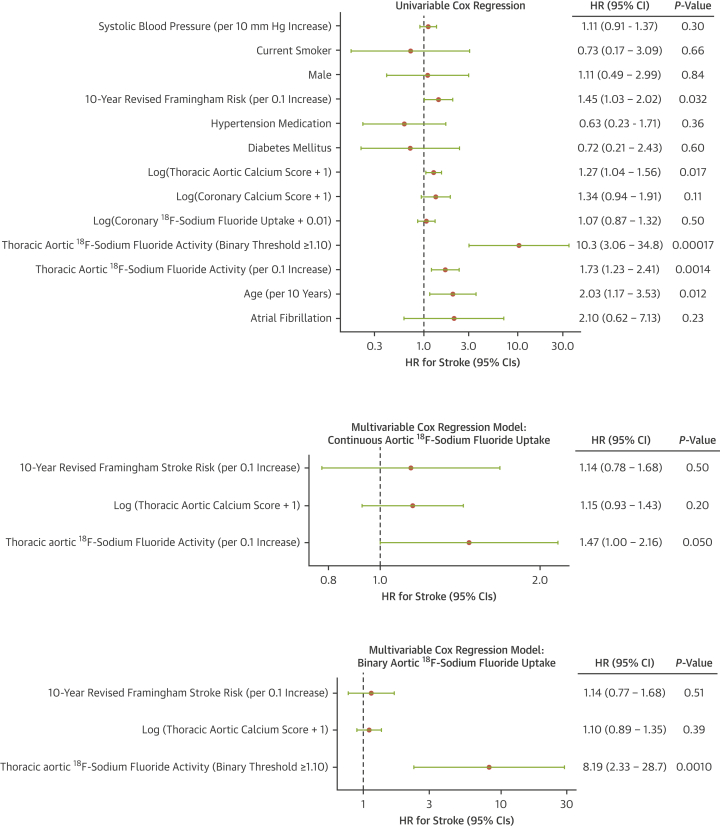
Predictors of Stroke Univariable Cox regression including clinical variables previously associated with stroke in the revised Framingham stroke risk score and imaging parameters. Multivariable Cox regression models including 10-year revised Framingham stroke risk, thoracic aortic calcium score, and thoracic aortic ^18^F-sodium fluoride activity, both as a continuous (middle plot) or binary (bottom plot) variable. Thoracic aortic ^18^F-sodium fluoride activity is the only variable associated with stroke in either multivariable model.

On univariable Cox regression, baseline thoracic aortic ^18^F-sodium fluoride activity, either continuous (HR: 1.73 [95% CI: 1.23-2.41] per 0.1 increase; *P =* 0.0014) or binary (high ≥1.1 or low <1.1; HR: 10.3 [95% CI: 3.06-34.8]; *P =* 0.00017), as well as baseline thoracic aortic calcium score (log[thoracic aortic calcium score + 1]: HR: 1.27 [95% CI: 1.04-1.56]; *P =* 0.017), 10-year revised Framingham stroke risk score (HR: 1.45 [95% CI: 1.03-2.02]; *P* = 0.032 per 0.1 increase), and age (HR: 2.03 [95% CI: 1.17-3.53] per 10 years; *P =* 0.012) were associated with ischemic stroke (Figure 4). Multivariable Cox regression models combining the variables associated with stroke on univariable regression demonstrated that thoracic aortic ^18^F-sodium fluoride activity was the only variable associated with stroke (continuous HR: 1.47 [95% CI: 1.00-2.16]; *P =* 0.050; c-statistic 0.71; binary HR: 8.19 [95% CI: 2.33-28.7]; *P =* 0.0010; c-statistic 0.78) (Figure 4, Supplemental Table 2).

## Discussion

This is the largest cohort of patients with cardiovascular disease undergoing prospective thoracic ^18^F-sodium fluoride PET-CT. We demonstrate, for the first time, that thoracic aortic ^18^F-sodium fluoride activity is associated with both thoracic aortic atheromatous plaque disease progression and a 10-fold increased future risk of ischemic stroke. Importantly, we found that regional ^18^F-sodium fluoride activity predicts disease progression and clinical events related to the vascular territory under evaluation. This relationship suggests that ^18^F-sodium fluoride PET-CT may have value in assessing disease activity in arterial conduit and major vessels as well as indicating risk of related future clinical ischemic events.

We have previously explored total coronary ^18^F-sodium fluoride activity using coronary ^18^F-sodium fluoride activity as a marker of global tracer activity across the coronary circulation. We demonstrated that a higher coronary ^18^F-sodium fluoride activity was associated with faster coronary disease progression and served as a powerful predictor of myocardial infarction, outperforming clinical risk scores and coronary artery calcium scores.^12^^,^^18^ This work led us to hypothesize that similar associations might apply to thoracic aortic atheroma and the risk of ischemic stroke. Given that the thoracic aorta is in the field of view of all of our previous prospective cardiovascular ^18^F-sodium fluoride PET-CT studies, we aimed to assess the relationship between aortic ^18^F-sodium fluoride activity, the progression of aortic calcification, and the risk of ischemic stroke. We were able to demonstrate that thoracic aortic ^18^F-sodium fluoride activity was associated with both progression of aortic atherosclerosis and subsequent ischemic stroke. This is consistent with prior work in other disease states that have found ^18^F-sodium fluoride activity to be indicative of disease activity, providing powerful prediction of disease progression and clinical events.^9^^,^^18^^,^^24^

We explored whether the relationship between ^18^F-sodium fluoride activity and cardiovascular events was specific to the vascular territory under evaluation. Would thoracic aortic ^18^F-sodium fluoride activity predict myocardial infarction, and would coronary ^18^F-sodium fluoride activity predict ischemic stroke? We observed that atherosclerotic ^18^F-sodium fluoride activity was specific to the circulation being assessed, with coronary ^18^F-sodium fluoride activity being able to identify those at risk of myocardial infarction but not stroke, whereas thoracic aortic ^18^F-sodium fluoride activity identified ischemic stroke risk but not the risk of myocardial infarction (Central Illustration). These findings are intuitive and plausible given that coronary plaque will not cause stroke and aortic plaque will not cause myocardial infarction. How might this relationship be used to improve risk stratification and targeted treatment strategies?

**Central Illustration:**
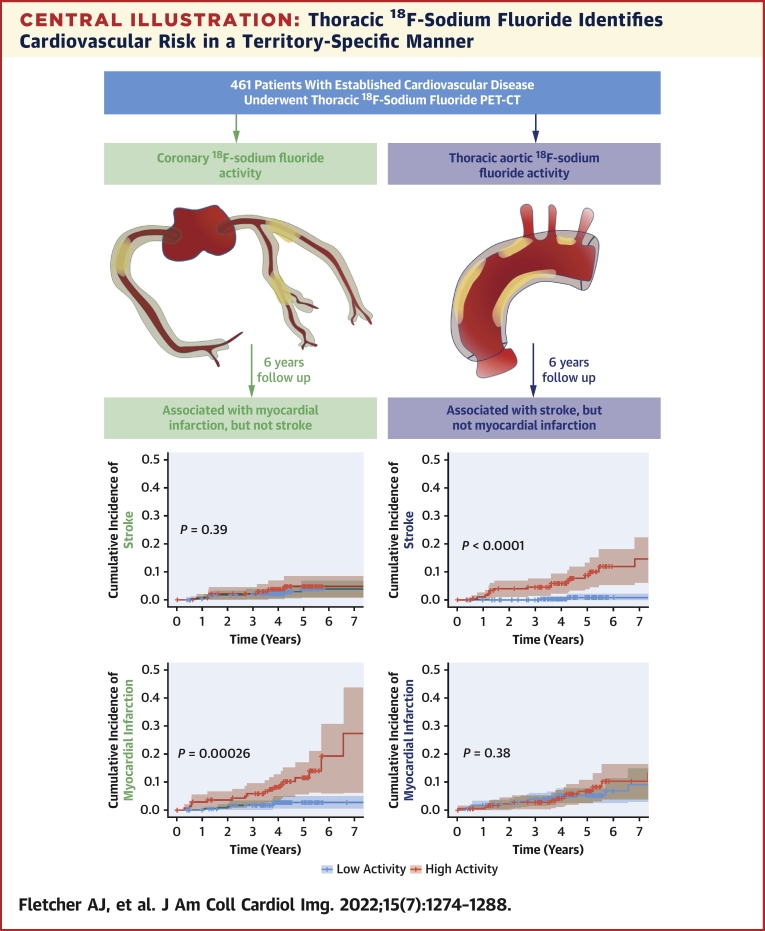
Thoracic ^18^F-Sodium Fluoride Identifies Cardiovascular Risk in a Territory-Specific Manner A total of 461 patients underwent thoracic ^18^F-sodium fluoride positron emission tomography and computed tomography (PET-CT), allowing the calculation of both thoracic aortic and coronary ^18^F-sodium fluoride activity. After a mean of 6 years, high thoracic aortic ^18^F-sodium fluoride was associated with ischemic stroke, but not myocardial infarction. High coronary ^18^F-sodium fluoride was associated with myocardial infarction but not stroke.

**Figure 5:**
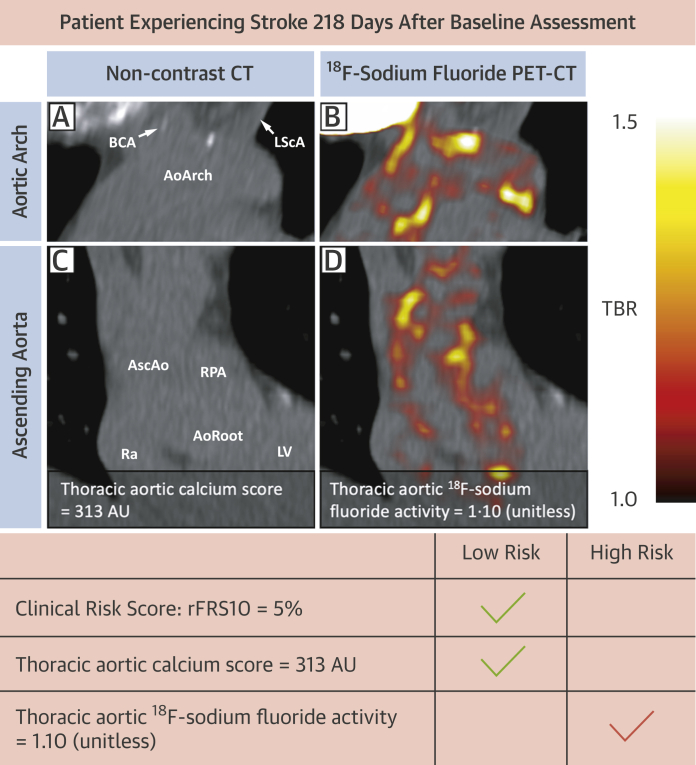
Example of Association Between ^18^F-Sodium Fluoride Positron Emission Tomography and Future Stroke Baseline noncontrast computed tomography (CT) and ^18^F-sodium fluoride positron emission tomography (PET) of the aortic arch **(A and B)** and ascending aorta **(C and D)**. A 60-year-old patient with hypertension and moderate aortic stenosis, on aspirin and clopidogrel. Overall low clinical risk score and thoracic aortic calcium scores, but high thoracic aortic ^18^F-sodium fluoride activity. Patient had bilateral posterior circulation infarcts at day 218 of follow-up with normal carotid arteries and no atrial fibrillation. AoArch = aortic arch; AoRoot = aortic root; AscAo = ascending aorta; AU = Agatston units; BCA = brachiocephalic artery; LScA = left subclavian artery; LV = left ventricle; Ra = right atrium; rFRS10 = Revised 10-year Framingham Stroke Risk Score; RPA = right pulmonary artery; other abbreviation as in Figure 1.

Current strategies to prevent future stroke involve identifying those at risk and targeting modifiable risk factors.^25^ Clinical risk scores, such as revised Framingham stroke risk, can identify those at highest risk but suffer from a lack of patient-level specificity: like many risk scores, most events occur in low-risk patients.^26^ The ability to detect the activity of thoracic aortic atheroma may help adjust future stroke risk profiles and potentially lead to better targeted treatment for patients at the highest risk (for an example see Figure 5).^27^ Previous work has identified that thoracic aortic calcium scores are associated with stroke risk independent of clinical factors.^7^^,^^8^^,^^28^^,^^29^ This is consistent with aortic atheroma proximal to the origins of the carotid and vertebral arteries representing a major source of stroke-related atherothrombotic emboli. Our work builds on this concept by incorporating biological disease activity and demonstrates that thoracic aortic ^18^F-sodium fluoride activity was associated with future stroke in addition to the known causes of stroke even after adjustment for clinical risk factors and thoracic aortic calcium scoring. From the clinical perspective, this indicates that thoracic ^18^F-sodium fluoride PET-CT can provide simultaneous assessment of both thoracic aortic and coronary ^18^F-sodium fluoride activity on a single scan, and thus can identify individuals at high risk of both stroke and myocardial infarction who might benefit from intensive or advanced preventative therapies. Larger studies in prospective cohorts are required to assess this further and are currently in progress (NCT02278211).

We set out to explore whether thoracic aortic atherosclerosis disease activity was associated with the future risk of atherothromboembolic clinical events. We therefore specifically examined for ischemic stroke events but excluded lacunar strokes, because of its presumed differing pathophysiology, as well as transient ischemic attacks, because there is often clinical uncertainty regarding these events and we did not wish to introduce unnecessary noise from the misclassification of events. Consequently, we restricted our analysis to patients with clinical presentations of ischemic stroke that are likely to be of atherothromboembolic origin. Although we were able to demonstrate a strong association with thoracic aortic ^18^F-sodium fluoride activity, we cannot be certain that these events were attributable to thromboembolism from aortic atheroma. Indeed, we accept that thoracic aortic ^18^F-sodium fluoride activity may also be indicative of disease activity within the head and neck vessels. Unfortunately, the head and neck arterial circulation was not within the field of view of our PET-CT images, and we cannot assess for the presence of such an association here. Moreover, nearly one-half of our patients experiencing stroke had competing risks, such as prior or new-onset atrial fibrillation. However, thoracic aortic ^18^F-sodium fluoride activity was high in patients with either carotid disease or atrial fibrillation, and aortic atheroma still represents a potential source of thromboembolism in these patients. Further, sensitivity analysis after excluding those with competing mechanisms for stroke demonstrated continuing superiority of thoracic aortic ^18^F-sodium fluoride activity over clinical risk scores and calcium scoring.

### Study limitations

It is important to highlight some further limitations of our work. We acknowledge that the overall number of stroke events is relatively small although the overall incidence rate of 9.2 per 1,000 patient-years is almost double that reported in a cohort of similar age and ethnicity, likely reflecting the enrichment and inclusion criteria of cardiovascular disease in our study cohort.^30^ Although the current work represent largest study assessing of thoracic aortic ^18^F-sodium fluoride PET, the small number of stroke events limits the robustness of our conclusions and requires further validation in bigger cohorts with larger numbers of events. We have combined 4 cohorts of patients with a combination of coronary artery disease and aortic stenosis representing a relatively heterogeneous cohort. Overall, combining these groups reflects a cohort of patients with prevalent cardiovascular risk factors, but the results may not be applicable to those with lower overall cardiovascular risk. Finally, while demonstrating an association between aortic ^18^F-sodium fluoride activity and stroke, the mechanism by which risk is conferred can only be notional, with some patients having competing risks for stroke. Large prospective studies assessing the relationship between thoracic aortic ^18^F-sodium fluoride activity and stroke, as well as coronary ^18^F-sodium fluoride activity and myocardial infarction, are now required to validate our findings in further external patient cohorts.

## Conclusions

We have found that high thoracic aortic ^18^F-sodium fluoride activity is correlated with thoracic aortic atherosclerotic disease progression and a 10-fold increased risk of future ischemic stroke. This relationship remained after adjustment for clinical risk factors, thoracic aortic calcium score, and the presence of alternative stroke causes. The predictive ability of ^18^F-sodium fluoride activity appears to be specific to the location of arterial ^18^F-sodium fluoride activity, with clinical events occurring in the arterial territory of increased activity. External validation in large cohorts is now required to establish whether there is a role for ^18^F-sodium fluoride PET in guiding treatment in a patient-specific manner.

Perspectives **COMPETENCY IN CLINICAL KNOWLEDGE:** Imaging assessments of arterial ^18^F-sodium fluoride activity predict atherosclerotic disease progression and the future risk of atherothrombotic events specific to the target vessel territory.

**TRANSLATIONAL OUTLOOK:** Both aortic and coronary ^18^F-sodium fluoride positron emission tomography can be quantified on thoracic ^18^F-sodium fluoride imaging and provide complementary information to computed tomography and cardiovascular risk scores related to disease activity and subsequent major adverse cardiovascular events.

## Funding Support and Author Disclosures

Drs Fletcher (FS/19/15/34155), Tzolos (FS/17/51/33096), Syed (FS/18/31/33676), Moss (AA/18/3/34220), Walker (FS/19/15/34155), Williams (FS/ICRF/20/26002), Newby (CH/09/002, RG/16/10/32375, RE/18/5/34216), and Dweck (FS/14/78/31020) are supported by the British Heart Foundation. Dr Lembo is supported by the International PhD programme in Cardiovascular Pathophysiology and Therapeutics (CardioPaTh). Dr van Beek is supported by SINAPSE. Dr Adamson is supported by a Heart Foundation of New Zealand Senior Fellowship (1844). Dr Wardlaw is supported by the UK Dementia Research Institute (funded by the MRC, Alzheimer Society and Alzheimer Research UK). Dr Slomka and FusionQuant Development are supported by the National Institute of Health Grant HL135557 (PI: Dr Slomka); and his laboratory is supported by National Institutes of Health R01HL135557. Dr Newby is the recipient of a Wellcome Trust Senior Investigator Award (WT103782AIA); and holds investigator-initiated research grants from Siemens Healthineers. Dr Dweck is the recipient of the Sir Jules Thorn Award for Biomedical Research 2015 (15/JTA). The Edinburgh Imaging facility QMRI (Edinburgh) is supported by the National Health Service Research Scotland through National Health Service Lothian Health Board. All other authors have reported that they have no relationships relevant to the contents of this paper to disclose.
