# Supplementary material for: Thoracic Aortic 18F-Sodium Fluoride Activity and Ischemic Stroke in Patients With Established Cardiovascular Disease
Source: JACC Cardiovasc Imaging. 2022 Jul;15(7):1274–88. doi: 10.1016/j.jcmg.2021.12.013 (PMC9252920; doi:10.1016/j.jcmg.2021.12.013)
Supplement: Supplemental Data [file mmc1.docx]

**Supplemental Methods**

**Study Populations**

Observational study in patients with stable coronary artery disease cohort: NCT01749254

Inclusion criteria for the cohort were patients with acute myocardial infarction or stable angina undergoing elective invasive coronary angiography. Exclusion criteria included age <50 years, insulin-dependent diabetes mellitus, women of childbearing age not receiving contraception, severe renal failure (serum creatinine >250 μmol/L) and known contrast allergy. Those with acute myocardial infarction were not included in the current analysis.

Randomized control trial in patients with stable coronary artery disease: NCT02110303

Inclusion criteria for the study were patients $\geq$40 years old with angiographically confirmed multivessel coronary disease defined as epicardial vessels with >50% stenosis or having undergone previous coronary revascularization. Exclusion criteria included acute coronary syndrome in the preceding 12 months, revascularization in the preceding 3 months, estimated glomerular filtration rate <30 mL/min/1.73m^2^, concurrent therapy with oral anticoagulants or thienopyridine (clopidogrel or prasugrel), or known allergy to iodine contrast media. Patients were randomized (1:1) to ticagrelor 90mg twice a day or placebo for a year.

Randomized controlled trial in patients with aortic stenosis: NCT02132026

Inclusion criteria for the study were patients >50 years old, with a peak aortic jet velocity of >2.5m/s as well as grade 2-4 aortic valve calcification on echocardiography. Exclusion criteria included planned aortic valve surgery, life expectancy <2 years, and long-term corticosteroid use and abnormalities of the esophagus/gastric emptying. Patients were randomized 2:1:2:1 to denosumab 60mg/6month injection, placebo injection, alendronic acid 70 mg/week or placebo tablet for 24 months.

Observational aortic stenosis cohort: NCT01358513

Inclusion criteria for the study were patients >50 years old with sclerosis, mild, moderate and severe stenosis as well as 20 controls. Exclusion criteria were those with insulin dependent diabetes, blood glucose >200 mg/dL and inability to undergo PET/CT. Those with sclerosis or controls were not included in the current analysis.

**Supplemental Results**

Randomized controlled trial in patients with stable coronary artery disease: NCT02110303

Out of 220 patients recruited, 201 (91%) participants had baseline ^18^F-NaF PET-CT of the entire thoracic aorta available for analysis. Average follow up period was 4·0±0·8 years

Observational stable coronary artery disease cohort: NCT01749254

Out of 40 patients assessed, 38 (95%) participants had baseline ^18^F-sodium fluoride (^18^F-NaF) positron emission tomography and computed tomography (PET-CT) of the thoracic aorta available for analysis (Supplementary Figure 2). Average follow up period was 8·0±1·7 years.

Randomized controlled trial in patients with aortic stenosis: NCT02132026

Out of 199 patients recruited, 158 (78%) participants had baseline ^18^F-NaF PET-CT of the entire thoracic aorta available for analysis. Average follow up period was 4·0±0·8 years.

Observational aortic stenosis cohort: NCT01358513

Out of 81 patients assessed, 63 (51%) had aortic stenosis with baseline 18F-NaF PET-CT of the entire thoracic aorta available for analysis. Average follow up period was 8·1±2·8 years.

The final study cohort consisted of 461 patients followed up for a mean of 6·1±2·3 years. All patients underwent comprehensive baseline assessment, including assessment of their cardiovascular risk profile. Revised Framingham stroke risk, a well validated clinical risk score for predicting the 10-year risk of stroke, was calculated in all patients, and was similar between cohorts (p=0·54, Table 1).^3^

**Supplemental Figures**

**Supplemental Figure 1**

**
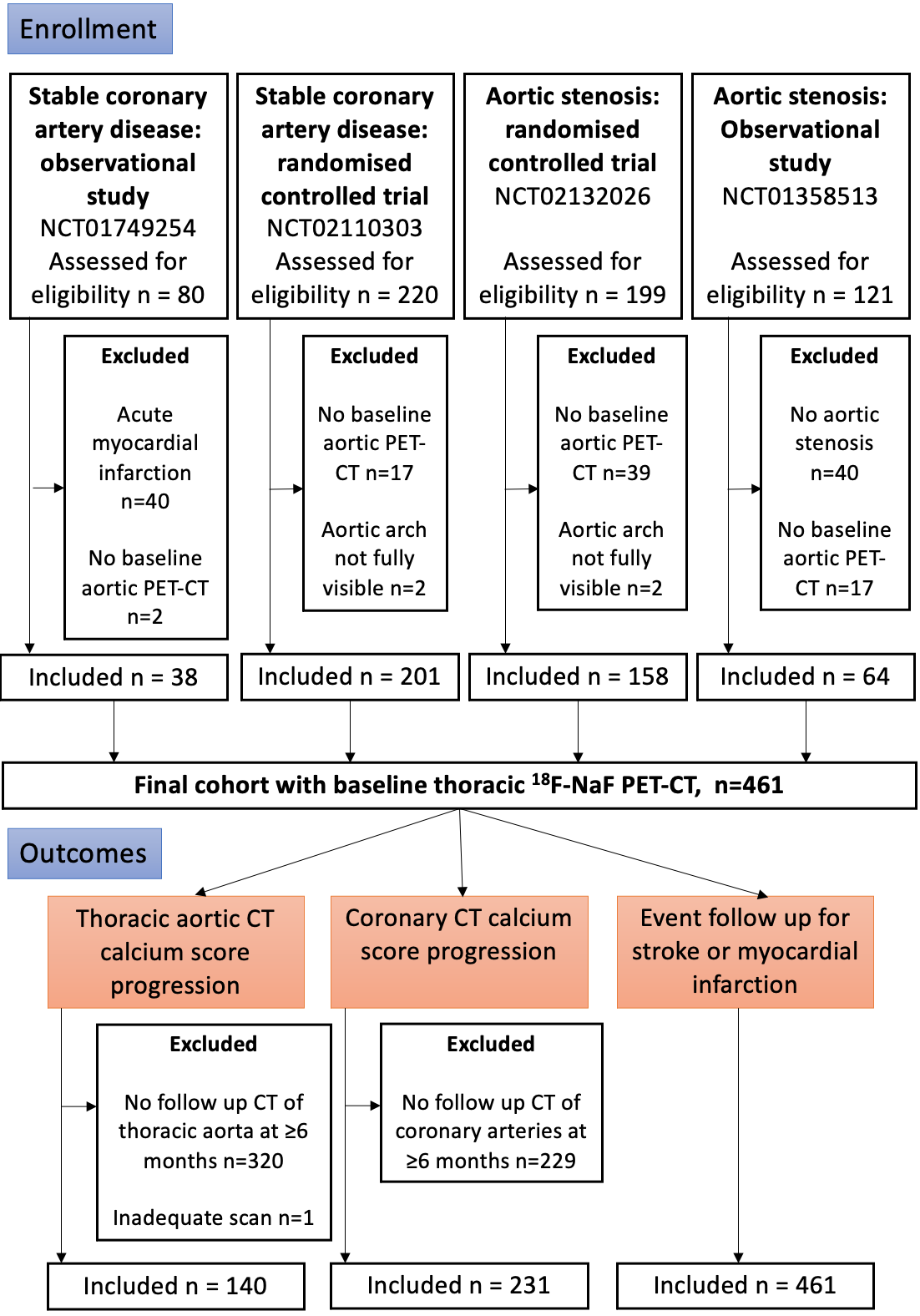
Study population CONSORT diagram**

**Supplemental Figure 2**

**Step-by-step method for calculating thoracic aortic atherosclerotic disease activity**

Step-by-step outline of measuring thoracic aortic atherosclerotic disease activity. (A) Co-register register 18F-sodium fluoride overlay to computed tomography image in three orthogonal planes using landmarks of the sternum, spine, and aortic wall (blue arrows). (B and C) Place a 2 cm^3^ region of interest in the center of the right (B) and left (C) atrium. The background activity is the cumulative SUV per cm^3^ from the volumes of interest in the left and right atrium. (D to F) With the ^18^F-sodium fluoride overlay turned off, a centerline function is used to draw the ascending aortic volume of interest in multiplanar reconstruction images. Perpendicular to the aorta, the volume of interest starts at the sinotubular junction (D) and finishes at the slice just proximal to the origin of the brachiocephalic artery (E). The width of the volume of interest is increased to the maximum ascending aortic diameter + 4 mm (F and H). The ^18^F-sodium fluoride overlay is reinstated to ensure good coverage (I). The ascending aortic atherosclerotic disease activity, and volume are calculated (I). The aortic arch volume of interest is drawn with the same method as the ascending aorta, starting with the slice immediately distal to the ascending aortic volume of interest (J), and finishing with the slice after the origin of the left subclavian artery (K). The width of the aortic arch volume of interest is increased to the maximal arch dimeter + 4 mm (M and N). The ^18^F-sodium fluoride overlay is reinstated to check good coverage and calculate the aortic arch atherosclerosis activity and volume (N). (O) Provides the formula for calculating overall thoracic aortic atherosclerosis activity, whilst (P) uses the values in the current case to provide a working example of thoracic aortic atherosclerosis activity calculation. Reproduced from Fletcher et al, under the terms of the Creative Commons CC BY license.^12^


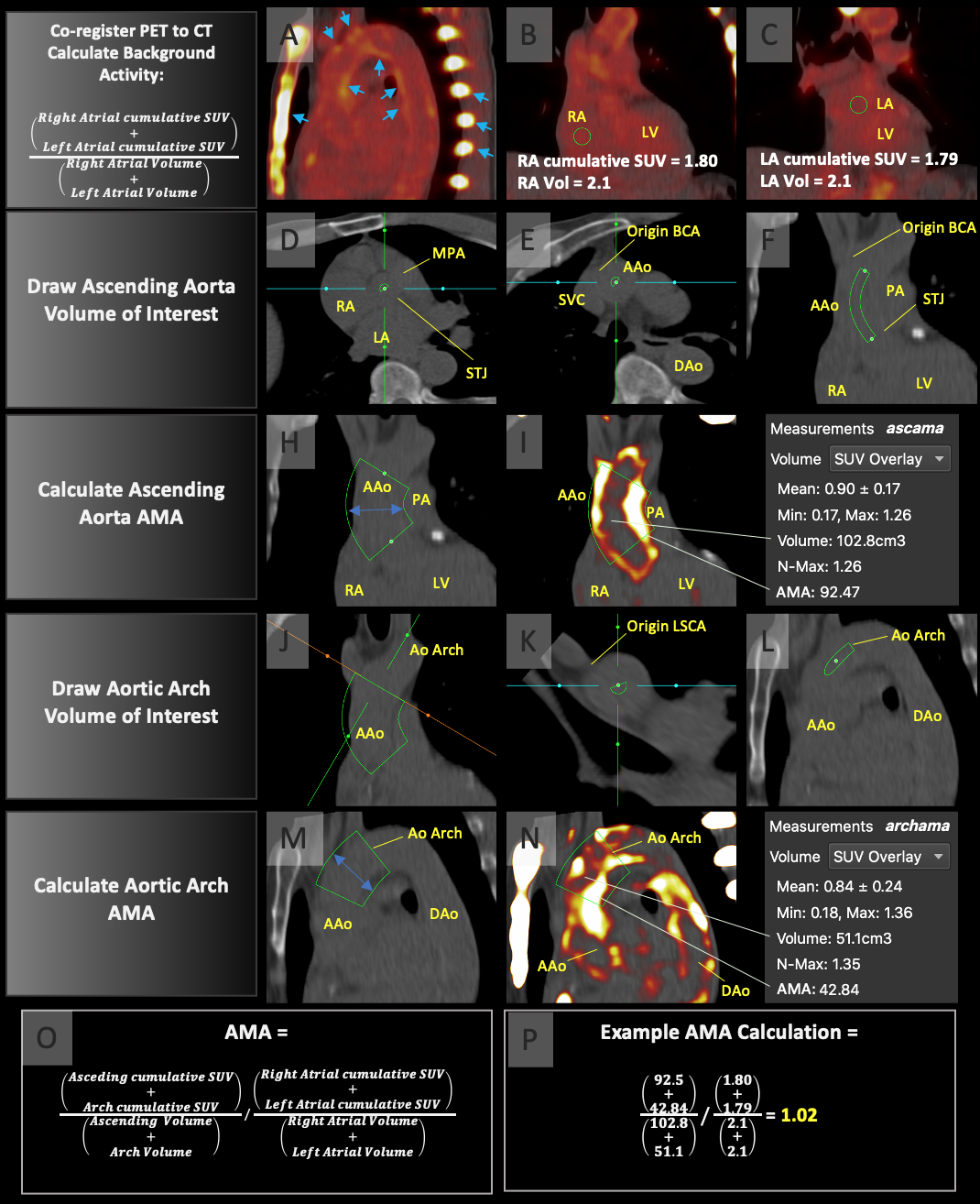


**Supplemental Figure 3**

**Time dependent area under the curve for prediction of stroke for imaging and clinical risk scores.**

Time-dependent area under the curve (AUC) for aortic ^18^F-sodium fluoride activity (light blue), thoracic aortic calcium score (blue), and 10-year Framingham stroke risk score (dark blue) over 5 years. P-values of analysis comparing AUC of aortic ^18^F-sodium fluoride activity with thoracic aortic calcium score (top row) and 10-year Framingham stroke risk (bottom row). Aortic ^18^F-sodium fluoride demonstrates high AUC which has an advantage in predictive ability over the first four years over thoracic aortic calcium scoring or 10-year Framingham stroke risk, but diminished by the 5^th^ year.


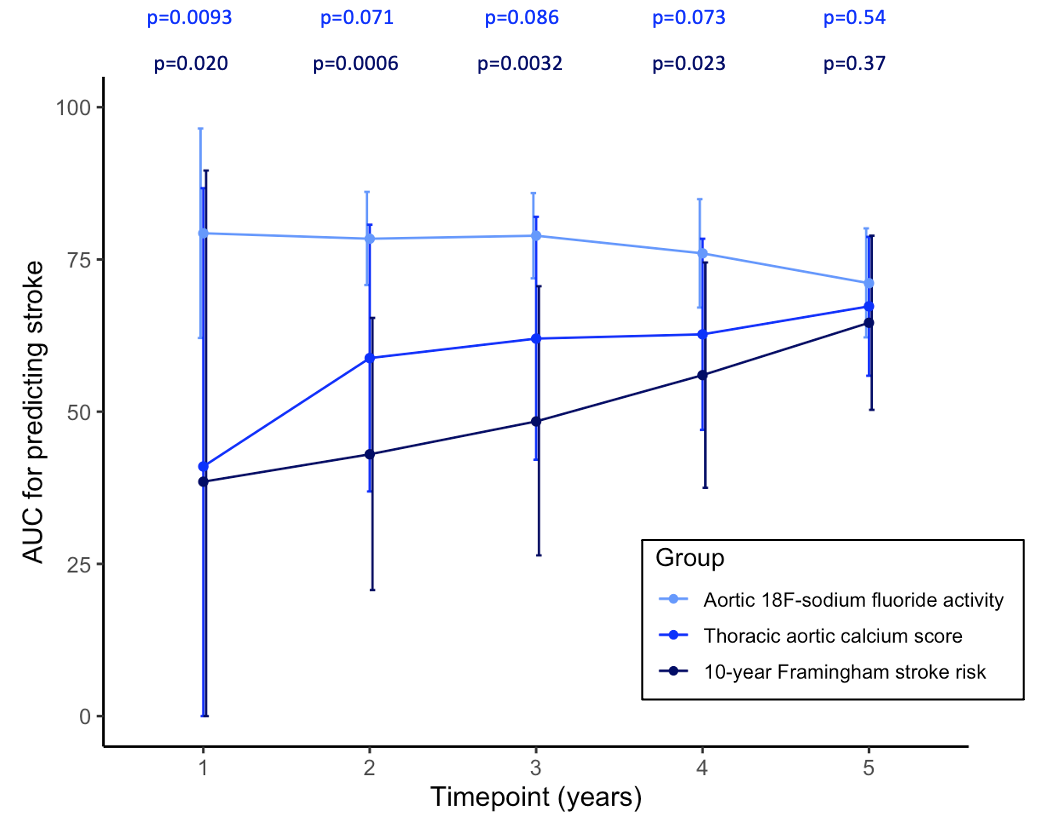


**Supplemental Figure 4**

**Sensitivity analysis with exclusion of patients with atrial fibrillation or stenotic carotid disease**

**
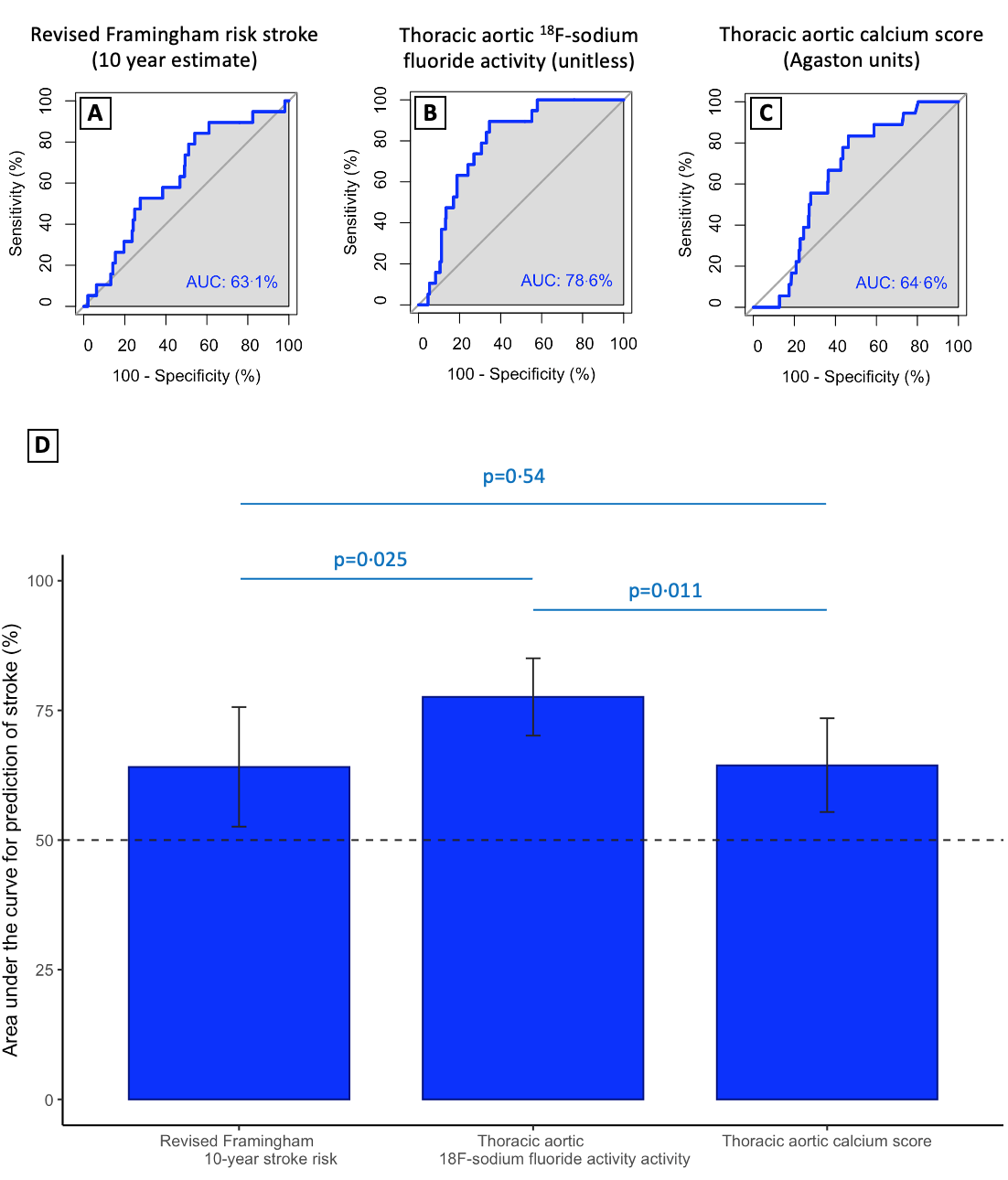
**Area under the curve for and sensitivity analysis for (**A**) thoracic aortic atherosclerotic disease activity, (**B**) 10-year stroke clinical risk score and (**C**) thoracic aortic calcium score. (**D**) Area under the curve analysis comparing aortic microcalcification activity, clinical risk score and thoracic aortic calcium score.

**Supplemental Tables**

**Supplemental Table 1**

**Clinical Features of patients experiencing territory ischemic stroke**

| Patient number | Age | Sex | Presenting symptoms | Clinical stroke type (Bamford classification) | 10-year revised Framingham stroke risk estimate (%) | Background | thoracic aortic ^18^F-sodium fluoride  activity | Carotid imaging | Brain imaging |
| --- | --- | --- | --- | --- | --- | --- | --- | --- | --- |
| D017 | 80 | F | Right dysphasia, hemianopia and hemiparesis | L PACS | 11 | Severe aortic stenosis, diabetes, hypertension | 1.12 | Not performed | CT - Acute infarction of the left insular cortex |
| D022 | 74 | M | Slurred speech and confusion | L POCS | 21 | New atrial fibrillation, hypertension | 1.11 | Not performed | MRI - Infarct in the left cerebellum |
| D042 | 69 | M | Glasgow coma scale 3, fatal | R POCS | 24 | Previous TIA, hypertension, right carotid endarterectomy | 1.15 | US - 90% stenosis right pre-operatively | CT - New right cerebellar hemisphere infarct |
| D073 | 70 | M | Left sided weakness, hemianopia and partial seizures | R PACS | 13 | New paroxysmal atrial fibrillation, hypertension | 1.18 | US - no significant stenosis | CT - No acute infarction detected |
| D120 | 61 | M | Left facial droop and dysphasia | R PACS | 18 | Diabetes, hypertension | 1.19 | US - no significant stenosis | CT - Infarct in area of right Sylvian fissure |
| SA09 | 84 | M | Right sided facial, leg and arm weakness with dysarthria | L PACS | 8 | Previous stroke, pacemaker, atrial fibrillation, on warfarin, diabetes, hypertension | 1.15 | US - no significant stenosis | CT - Left internal capsule infarct |
| SA10 | 73 | M | Sudden onset ataxia | L POCS | 24 | Diabetes, hypertension | 1.16 | CT - normal carotids | CT - Left vertebral artery occlusion and medullary infarct |
| SA35 | 75 | M | Left sided weakness facial droop and dysarthria | R PACS | 14 | Previous stroke, atrial fibrillation on apixaban, diabetes, hypertension | 1.15 | Not performed | CT - No acute infarct detected |
| SA37 | 73 | M | Left sided facial droop and weakness | R PACS | 29 | Previous stroke, hypertension | 1.15 | US - right internal carotid stenosis | CT - Right MCA territory infarct |
| AS2002 | 83 | M | Sudden ataxia with wide-based gate | Bilat POCS | 24 | Hypertension, previous MI, smoker, previous TAVI, paroxysmal AF on Apixaban | 1.19 | Not performed | CT - Bilateral cerebellar cortical infarcts |
| AS2097 | 76 | M | Left arm weakness and slurred speech | R POCS | 22 | Hypertension, NIV for kyphoscoliosis, previous TIA | 1.05 | US - no significant disease | CT - Established right parietal infarct |
| AS2110 | 58 | M | Right sided visual field loss | L POCS | 20 | Aortic stenosis | 1.11 | US - no significant stenosis | CT - Left occipital lobe infarction |
| AS2112 | 69 | M | Right sided hand weakness and numbness with visual field loss | L PACS | 14 | Hypertension, angina, heart failure, atrial fibrillation, peripheral vascular disease, high cholesterol | 1.05 | Not performed | MRI - Small cortical infarct left frontal lobe |
| AS2146 | 65 | M | No local notes | R PACS | 20 | Aortic stenosis | 1.21 | No local scan performed | No local scan performed |
| AS2164 | 69 | F | Right sided dysphasia and weakness | L PACS | 30 | Hypertension, Diabetes, smoker, high cholesterol | 1.13 | Not performed | CT - left and right occipital infarct |
| AS2182 | 60 | M | Sudden onset vision loss and ataxia | Bilat POCS | 5 | Hypertension, aortic stenosis | 1.10 | CT with contrast - no significant stenosis | MRI - Sub-acute bilateral POCS affecting both thalami and midbrain |
| R04 | 77 | M | Dizziness and unsteady gait | Bilat POCS | 22 | Atrial fibrillation on apixaban, hypertension | 1.09 | Not performed | CT - probable bilateral cerebellar cortical infarcts |
| R013 | 84 | F | Confusion, slurred speech, right sided facial and upper limb weakness | L PACS | 23 | Hypertension, high cholesterol, aortic stenosis | 1.26 | Significant stenosis of right carotid | CT - acute MCA perforator territory infarct |
| R024 | 80 | F | Slurred speech and left facial droop | L PACS | 38 | Atrial fibrillation on warfarin, hypertension, previous stroke, high cholesterol | 1.19 | Not performed | CT - no acute stroke identified |
| R044 | 80 | F | Found collapsed | R TACS | 16 | Hypertension, high cholesterol | 1.15 | Not performed | CT - massive right cerebral hemisphere ischemic stroke |
| R090 | 81 | M | Expressive dysphasia, slurred speech, right facial and limb weakness | L PACS | 19 | Atrial fibrillation on warfarin, diabetes, previous stroke | 1.17 | US - no significant stenosis | CT - no acute stroke identified |
| R097 | 79 | M | Collapse | R PACS | 20 | Hypertension, high cholesterol, ex smoker | 1.24 | Not performed | CT - small right parietal cortical infarct |
| R103 | 79 | F | Ataxia, visual impairment | L POCS | 14 | Hypertension, high cholesterol | 1.19 | Not performed | CT - subacute left cerebellar infarct |

**Supplemental Table 2**

Multivariate Cox models using a combination of factors significantly associated with time to stroke on univariable analysis and aortic ^18^F-sodium fluoride activity as either a continuous variable (model 1) or binary variable (model 2)

| **Model Number** | **Hazard ratio (95% confidence interval)** | **p-value** | **Concordance** |
| --- | --- | --- | --- |
| **Model 1**  Thoracic aortic ^18^F-sodium fluoride activity (per 0.1 increase)  Log(Thoracic aortic calcium score+1)  10-year revised Framingham stroke risk (per 0.1 increase) | 1.47 (1.00 to 2.16)  1.15 (0.93 to 1.43)  1.14 (0.77 to 1.68) | 0.050  0.20  0.50 | 0.71 |
| **Model 2**  Thoracic Aortic ^18^F-sodium fluoride activity (>1.10 unitless)  Log(Thoracic aortic calcium score+1)  10-year revised Framingham stroke risk (per 0.1 increase) | 8.19 (2.33 to 28.72)  1.10 (0.89 to 1.35)  1.14 (0.77 to 1.68) | 0.0010  0.51  0.39 | 0.78 |
